# Disproportionate Risk of Cancer Following Diagnosis of Peripheral Artery Disease

**DOI:** 10.1016/j.jacadv.2026.102889

**Published:** 2026-06-15

**Authors:** Richard A. Baylis, Allen Haas, Hua Gao, Sharon H. Giordano, Kevin T. Nead, Nicholas J. Leeper

**Affiliations:** a Division of Cardiology, Department of Medicine, University of California San Francisco, San Francisco, California, USA; b Department of Health Services Research, University of Texas M.D. Anderson Cancer Center, Houston, USA; c Stanford Cardiovascular Institute, Stanford, California, USA; d Division of Vascular Surgery, Department of Surgery, Stanford University School of Medicine, Stanford, California, USA; e Department of Breast Medical Oncology, University of Texas M.D. Anderson Cancer Center, Houston, USA; f Department of Epidemiology, University of Texas M.D. Anderson Cancer Center, Houston, USA; g Department of Breast Radiation Oncology, University of Texas M.D. Anderson Cancer Center, Houston, USA; h Division of Cardiovascular Medicine, Department of Medicine, Stanford University School of Medicine, Stanford, California, USA

**Keywords:** cancer, cardio-oncology, peripheral artery disease

## Abstract

**Background:**

Cancer and cardiovascular disease (CVD) are leading causes of death and share multiple risk factors. Population-level and mechanistic studies support a causal association between CVD and cancer. Recent preclinical studies suggest that peripheral arterial disease (PAD) may confer unique cancer risks. However, the specific contribution of PAD to cancer incidence—and whether this risk exceeds that of other CVD types—remains unclear.

**Objectives:**

The objective of the study was to test whether PAD is an independent risk factor for cancer and whether it is associated with a greater cancer risk than other forms of CVD.

**Methods:**

We conducted a retrospective cohort study using the MarketScan Research Database (2013-2023). Adults aged ≥40 years with ≥36 months of continuous coverage were classified as having PAD, non-PAD CVD, or no CVD. The primary outcome was time to incident cancer, analyzed using an inverse probability of treatment-weighted Fine-Gray subdistribution hazard model adjusted for shared risk factors.

**Results:**

Our cohort included 87,594 individuals with PAD, 917,680 with non-PAD CVD, and 4,303,899 without CVD. Multivariable-adjusted inverse probability of treatment-weighted regression controlling for shared risk factors demonstrated that patients with PAD had a greater risk of cancer than individuals with non-PAD CVD (subdistribution HR: 1.027; 95% CI: 1.001-1.053; *P* = 0.044) and those without CVD (subdistribution HR: 1.158; 95% CI: 1.129-1.187; *P* < 0.0001). Individual analysis of the 20 most common cancers demonstrated associations of PAD with specific malignancies.

**Conclusions:**

Patients with PAD experience a higher risk of multiple forms of cancer after controlling for shared risk factors.

Cancer and cardiovascular disease (CVD) are extending their dominance as the principle causes of global mortality and, given their prevalence, are commonly found coincident in aging patients. Historically, this coexistence has been explained by shared risk factors (eg, smoking). However, emerging literature supports a role for CVD in the development of cancer. This association has been established in recent epidemiologic studies, which have shown that patients diagnosed with CVD experience higher rates of cancer diagnosis even after controlling for shared risk factors.^1^, ^2^, ^3^ Preclinical mechanistic studies of CVD using experimental myocardial infarction (MI), heart failure, and peripheral ischemia models have proposed roles for circulating factors, myelopoiesis, and systemic inflammation as pathologic cancer drivers.^4^, ^5^, ^6^, ^7^, ^8^, ^9^ Interestingly, the risk of cancer development has been found to vary based on the type of CVD.^3^ Although the presence of either atherosclerotic CVD or a nonatherosclerotic form significantly increased the rate of cancer development, the presence of atherosclerotic CVD was associated with a particularly enhanced risk, suggesting that the pathogenesis of atherosclerosis may be uniquely oncogenic.

Atherosclerosis can manifest clinically in manifold ways. The cardiovascular mortality from atherosclerosis is primarily driven by complications of coronary artery disease (CAD) whereas atherothrombotic narrowing of peripheral arteries, defined as peripheral arterial disease (PAD), contributes to significant morbidity ranging from claudication to limb threatening ischemia. However, despite being a relatively minor direct cause of mortality, it has been well documented that a diagnosis of PAD significantly increases premature mortality from both CVD (primarily concomitant CAD)^10^ but also non-CVD causes. Indeed, in the EUCLID study of ticagrelor in symptomatic PAD patients, of the 9% of patients that died during 30 month median follow-up, 41% of deaths were of non-CVD origin, including 18% from malignancy.^11^ Furthermore, a recent preclinical study found that a murine model of hind limb ischemia (HLI) augmented tumor growth secondary to immune modulation.^2^ Here we test the hypothesis that a diagnosis of PAD is associated with an increased risk of cancer.

## Methods

### Data source

We conducted a retrospective cohort study using the Merative MarketScan Research Database, which contains deidentified data for approximately 161 million patients, including enrollment records and health insurance claims from inpatient services, outpatient visits, and outpatient prescription drugs. We limited our analyses to the 111,320,535 patients on plans that report mortality information. Demographic data for patients with and without mortality data are presented in Supplemental Table 1. This study followed the Strengthening the Reporting of Observational Studies in Epidemiology (STROBE) reporting guidelines for cohort studies and was exempted by the University of Texas MD Anderson Cancer Center Institutional Review Board because of its use of deidentified data.

### Cohort selection

**Figure 1:**
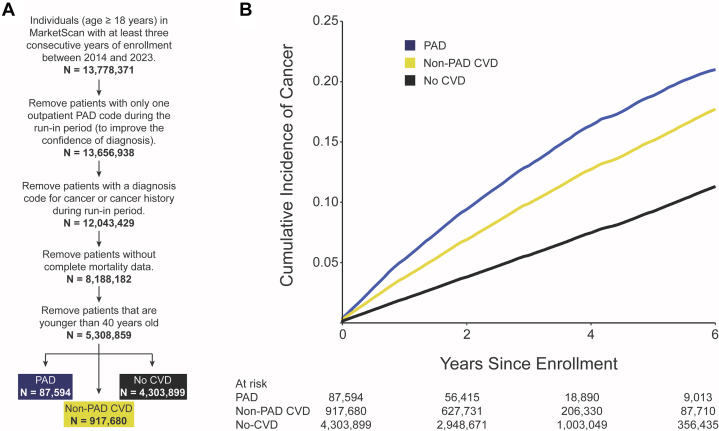
Cumulative Incidence for All Cancers by PAD Status A. Flow diagram showing sequential inclusion criteria for group derivation. B. Cumulative incidence from index date to the diagnosis of any cancers among the cohorts. CVD = cardiovascular disease; PAD = peripheral artery disease.

We identified patients at least 40 years of age with at least 36 months of continuous coverage between 2013 and 2023. We included a 24-month run-in period before the start of follow-up during which patients were classified into 1 of 3 groups in preferential order: individuals with PAD (PAD); individuals with non-PAD CVD (non-PAD CVD); and individuals without any CVD (no CVD). International Classification of Disease (ICD)-9th (ICD-9) or -10th (ICD-10) edition diagnosis or Current Procedural Terminology codes were used to define diseases states, including comorbidities, and are detailed in Supplemental Table 2.^1^ The diagnosis of PAD was defined as: 1) 2 or more ICD-9 or ICD-10 Revision, codes from an outpatient setting on separate dates; 2) 1 inpatient code ICD code; or 3) 1 procedure code, for PAD. Tobacco use was determined using the following ICD codes (ICD-9) 305.1, 649.0x, 989.84, V15.82 and (ICD-10) F17.200, 099.33, T65.2, Z87.891. In accordance with this definition, patients with only 1 outpatient code for PAD were excluded due to their unclear PAD status. Patients were additionally required to have mortality data either from linking to the social security administration’s death master file or the MarketScan contributor’s enrollment information. The cohort selection workflow is presented in Figure 1.

### Quantitative bias analysis

To evaluate the potential impact of misclassified tobacco use on the observed associations we conducted a quantitative bias analysis by calculating confounding risk ratios and subsequently adjusted HRs and 95% CIs.^12^ We used published data to derive tobacco use prevalence and magnitudes of association of tobacco use with cancer.^13^, ^14^, ^15^

### Statistical analysis

Baseline characteristics for the unweighted cohort were compared using a chi-square test. The primary outcome was defined as the time from the index date (defined as 2 years from the start of coverage) to the diagnosis any incident cancer (event), death (competing risk), or end of coverage (censor). Secondary outcomes were time to incident cancer for each of the 20 most common cancers individually. Nonmelanoma skin cancers, including basal cell and squamous cell carcinomas, were not considered. Participants were censored in the individual cancer analyses when they developed other organ-specific cancers. We used an inverse probability of treatment-weighted (IPTW) Fine-Gray subdistribution hazard model to evaluate the association of PAD status on cancer risk, compared with individuals with non-PAD CVD or no CVD, with a competing risk of death. The purpose of the IPTW method is to evaluate the association of PAD with cancer risk as if the groups had been randomly assigned. The weights were calculated using a logistic model for PAD adjusted for age as a continuous variable and categorically for sex, diabetes, hypertension, chronic kidney disease, hyperlipidemia, and tobacco use. Standardized mean differences before and after weighting can be found in Supplemental Table 3 and the balance of covariates between the 3 main clinical cohorts is in Supplemental Table 4. We included the same covariables used to construct the IPTW in our regression model to better account for confounding. Proportional subdistribution hazards were assessed using a time-dependent interaction term. We considered a 2-sided *P* value of <0.05 to be statistically significant unless otherwise indicated. All analyses were performed using SAS Enterprise Guide (version 7.11), and R (R Foundation for Statistical Computing, version 4.2.1).

## Results

Our analytic cohort included 87,594 patients with PAD, 917,680 patients with non-PAD CVD and 4,303,899 patients without a diagnosis of CVD (no CVD). See Figure 1A for patient selection details. Baseline characteristics of the cohort before weighting are summarized in Table 1. As expected, patients with PAD were older with higher rates of cardiovascular risk factors including diabetes, hypertension, hyperlipidemia, chronic kidney disease, and smoking (Table 1). The overall median follow-up time was 31 months (IQR: 16-42 months) with subgroup follow-up information included in Supplemental Table 5.

**Table 1:** Unweighted Baseline Cohort Characteristics

	PAD Cases (n = 87,594)	CVD Controls (n = 917,680)	No CVD Controls (n = 4,303,899)	*P* Value
Age, y (mean ± SD)	72.6 ± 12.5	63.4 ± 12.3	54.6 ± 9.4	
Sex				<0.0001
Male	43,695 (49.9%)	467,313 (50.9%)	2,038,038 (47.4%)	
Female	43,899 (50.1%)	450,367 (49.1%)	2,265,861 (52.6%)	
Diabetes				<0.0001
No	38,323 (43.8%)	640,644 (69.8%)	3,706,446 (86.1%)	
Yes	49,271 (56.2%)	277,036 (30.2%)	597,453 (13.9%)	
Hypertension				<0.0001
No	10,275 (11.7%)	249,173 (27.2%)	2,899,807 (67.4%)	
Yes	77,319 (88.3%)	668,507 (72.8%)	1,404,092 (32.6%)	
Hyperlipidemia				<0.0001
No	17,365 (19.8%)	270,082 (29.4%)	2,782,892 (64.7%)	
Yes	70,229 (80.2%)	647,598 (70.6%)	1,521,007 (35.3%)	
CKD				<0.0001
No	72,986 (83.3%)	862,145 (93.9%)	4,264,433 (99.1%)	
Yes	14,608 (16.7%)	55,535 (6.1%)	39,466 (0.9%)	
ICD smoking				<0.0001
No	66,561 (76.0%)	778,992 (84.9%)	4,039,104 (93.8%)	
Yes	21,033 (24.0%)	138,688 (15.1%)	264,795 (6.2%)	
Insurance				<0.0001
HMO	5,581 (6.4%)	72,426 (7.9%)	384,905 (8.9%)	
PPO	41,009 (46.8%)	495,250 (54.0%)	2,427,927 (56.4%)	
Other	40,119 (45.8%)	338,701 (36.9%)	1,439,900 (33.5%)	
Unknown	885 (1.0%)	11,303 (1.2%)	51,167 (1.2%)	
Region				<0.0001
Northeast	24,497 (28.0%)	212,046 (23.1%)	771,840 (17.9%)	
North Central	29,805 (34.0%)	247,917 (27.0%)	990,698 (23.0%)	
South	27,503 (31.4%)	363,498 (39.6%)	1,873,165 (43.5%)	
West	5,634 (6.4%)	91,868 (10.0%)	653,714 (15.2%)	
Unknown	155 (0.2%)	2,351 (0.3%)	14,482 (0.3%)	

Values are n (%).

Baseline characteristics for the unweighted cohort were compared using a chi-square test.

CKD = chronic kidney disease; CVD = cardiovascular disease; HMO = health maintenance organization; ICD = International Classification of Disease; PAD = peripheral artery disease; PPO = preferred provider organization.

We examined the cumulative incidence of cancer in each IPTW group with death as a competing risk. We found a higher cancer risk among individuals with PAD compared to those with non-PAD CVD or no CVD (Figure 1B). The 5-year cumulative incidence of cancer was 18.84% (18.47%-19.20%) in participants with PAD, 15.11% (15.00%-15.23%) in participants with non-PAD CVD, and 9.24% (9.19%-9.28%) in participants without CVD. The absolute excess risk of PAD compared to no CVD is 9.6% and compared to non-PAD CVD is 3.7%.

Next, we implemented IPTW Fine-Gray subdistribution multivariable adjusted hazard models to evaluate the association of PAD status on cancer risk while accounting for competing risks and balancing baseline differences between groups (Table 2). Patients with PAD had higher rates of cancer diagnosis when compared to patients without CVD (subdistribution HR [SHR]: 1.158; 95% CI: 1.129-1.187; *P* < 0.0001). Interestingly, patients with PAD experienced a modest but statistically higher rate of cancer diagnosis when compared to nonPAD CVD (SHR: 1.027; 95% CI: 1.001-1.053; *P* = 0.0444), suggesting that PAD may have a larger influence on cancer diagnosis than other forms of CVD. We performed additional subgroup analyses comparing patients with PAD to those with CAD as well as those with heart failure. Patients with CAD experienced a similar rate of cancer development compared to those with PAD (SHR: 0.992; 95% CI: 0.964-1.021; *P* = 0.5823) whereas PAD was associated with a significantly increased cancer rate compared to heart failure (SHR: 1.073; 95% CI: 1.044-1.102; *P* < 0.0001). We observed statistically significant evidence of nonproportionality when modeling a time-dependent interaction term (*P* < 0.05).

**Table 2:** Multivariable Adjusted Risk of Cancer According to IPTW PAD Group

	HR (95% CI)	*P* Value
PAD vs No CVD	1.158 (1.129-1.187)	<0.0001
PAD vs CVD	1.027 (1.001-1.053)	0.0444
PAD vs CVD (smoker’s only)	1.209 (1.126-1.298)	<0.0001
PAD vs CAD	0.992 (0.964-1.021)	0.5823
PAD vs HF	1.073 (1.044-1.102)	<0.0001

Groups were compared using an inverse probability of treatment-weighted (IPTW) Fine-Gray subdistribution hazard model with a competing risk of death.

CAD = coronary artery disease; HF = heart failure; other abbreviations as in Table 1.

Smoking is a strong risk factor for both PAD and cancer and a critical variable in this analysis. Determination of smoking status using claims-based data with ICD codes has previously been shown to have a low sensitivity but very high specificity. Therefore, we performed an analysis that included only patients that smoked. Consistent with the original analysis, those patients with a smoking history and PAD had a significant increase in their risk of cancer when compared to patients with a smoking history and non-PAD CVD (SHR: 1.209; 95% CI: 1.126-1.298; *P* < 0.0001) (Table 2). Quantitative bias analysis using confounding risk ratios suggested that misclassification of tobacco use in claims data is unlikely to fully explain the observed association between PAD vs no-CVD or PAD vs heart failure, but may explain a significant proportion of the association between PAD vs non-PAD CVD (Supplemental Table 6).

**Figure 2:**
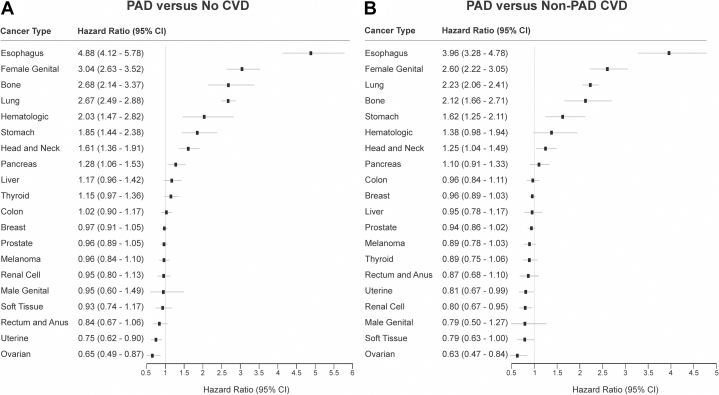
HRs Reflecting the Impact of PAD on Specific Cancers A. Individuals with peripheral artery disease (PAD) vs individuals without cardiovascular disease (CVD). B. HRs for specific cancers: individuals with PAD vs individuals with non-PAD CVD.

Finally, to determine if specific cancer risk was uniquely associated with PAD, the risk of the 20 most incident cancers in our cohort was assessed (Figure 2A). Compared to individuals without CVD, PAD patients experienced significantly increased risk for 8 cancers. When PAD patients were compared to patients with non-PAD CVD, patients with PAD experienced statistically significantly higher rates of esophageal, female genital, lung, bone, stomach, and head and neck cancers (Figure 2B).

## Discussion

This large retrospective cohort study investigating the incidence of cancer among patients with PAD demonstrates that patients with PAD experience significantly higher rates of cancer diagnoses when compared to patients without CVD and to those with non-PAD CVD that persists after controlling for shared risk factors. Patients with PAD experienced significantly higher rates of multiple cancer subtypes, highlighting the need for enhanced awareness and adherence to cancer screening guidelines in this population (Central Illustration).

**Central Illustration:**
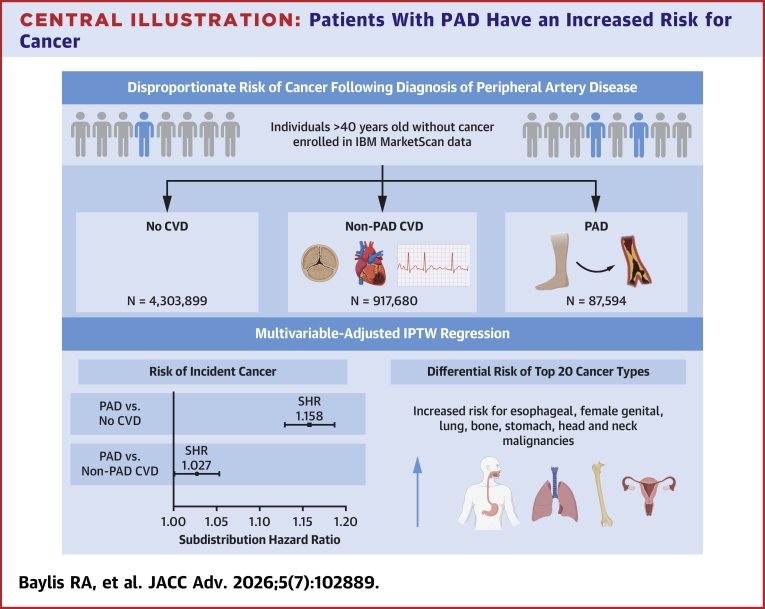
Patients With PAD Have an Increased Risk for Cancer In this large retrospective analysis of insurance claims data, patients with PAD were compared to patients with non-PAD CVD and no known CVD. After controlling for known confounding variables that might influence the development of cancer, patients with PAD were found to have a significantly elevated risk of incident cancer which appeared to be driven by increases in esophageal, female genital, lung, bone, stomach, and head and neck cancers. Illustrations crafted in BioRender. CVD = cardiovascular disease; IPTW = inverse probability of treatment-weighted; PAD = peripheral artery disease; SHR = subdistribution HR.

Our study builds on prior literature suggesting increased cancer incidence among patients with PAD. A retrospective study from 2020 analyzed data from the second largest German insurance fund, which included 96,528 patients with symptomatic PAD, and compared the incidence of cancer to the general German population.^16^ They found a significant increase in lung, bladder, pancreas, and colon cancer. They did not control for shared risk factors like smoking but suggested that shared risk factors likely played an important role in driving their observations. Indeed, large registry studies like the REACH registry have demonstrated that patients with PAD have a significantly higher burden of cardiovascular risk factors than patients with non-PAD CVD, many of which have previously been shown to increase the risk of cancer as well.^13^

A separate study attempted to control for the confounding influence of shared risk factors by analyzing patients in the Atherosclerosis Risk in Communities Study.^17^ They categorized the 13,106 participants without a pre-existing cancer diagnosis into symptomatic PAD, asymptomatic PAD, and five different ankle-brachial index categories, which were used to diagnose and determine the severity of PAD. They investigated 7 common cancer subtypes including bladder, breast, lung, colorectal, liver, pancreatic, and prostate cancer. Their study benefited from a long median follow-up of 25.3 years during which patients with symptomatic PAD experienced higher rates of new cancer diagnoses than those with asymptomatic PAD and those with a normal ankle-brachial index. They were limited by comparatively small cohorts and were only able to resolve a significant increase in lung cancer. Given the size of our cohort, we were able to resolve an increased risk for multiple cancer subtypes and, by demonstrating that the risk remains elevated even after controlling for shared risk factors, our data support the hypothesis that the presence of PAD may play an important role in driving cancer incidence across numerous cancer subtypes.

Although the retrospective nature of this manuscript cannot establish causality, it adds to an emerging body of literature that implicates atherogenesis in cancer development.^6^^,^^9^ Experimental studies show that MI–induced heart failure accelerates intestinal tumor progression,^6^ that early cardiac hypertrophy promotes tumor development and metastasis through direct cardiovascular-cancer signaling,^8^ and that MI-driven immune alterations create an immunosuppressive state conducive to breast cancer growth and dissemination.^4^

Atherosclerosis is a systemic vascular disease and the presence of plaque accumulation in 1 vascular bed implies there is likely disease in other territories. Although PAD and CAD are both characterized by the accumulation of atherosclerotic vascular debris, their pathogenesis has notable differences that may explain their differing predilection to various cancer subtypes.^18^ A unique aspect of PAD especially among symptomatic patients, compared to other forms of atherosclerotic disease, is the longstanding tissue ischemia to the lower extremities. This pathophysiology was the focus of an important recent preclinical study which sought to determine if the increased tumor growth seen after experimental MI in mice was due to direct cardiac injury or the result of tissue ischemia.^4^^,^^9^ The study performed HLI, or a sham procedure, in mice injected with a breast cancer cell line. The tumors in the mice that had undergone HLI grew significantly faster, which the authors associated with a myeloid shift in the blood and tumor immune cell infiltrate. Their data indicated that peripheral ischemia caused epigenetic changes in the bone marrow, which subsequently promoted tumor growth in mice that received bone marrow transplants from those subjected to HLI. Notably, similar changes in myelopoiesis have been seen in patients with PAD^19^ and it is likely that these hematopoietic effects of peripheral ischemia contribute to the increased cancer incidence observed in our study. In their HLI model, the vascular remodeling results in complete restoration of hind limb blood flow within several weeks, which suggests that elevated cancer risk may persist even after successful revascularization in patients.

Genetic associations provide an additional possible explanation for the cancer risk differences observed in PAD vs other forms of atherosclerotic CVD. Klarin^20^ performed a genome wide association study in patients from the Million Veteran Project and found 19 variants significantly associated with PAD. Many of these variants identified genes also indicated in CAD genome wide association study including *LDLR*, *LPA*, and *LPL*. However, there were multiple variants that were unique to PAD and informative to its specific pathogenesis including in *CHRNA3* (cholinergic receptor nicotinic alpha-3), which is strongly predictive of nicotine dependence, as well as *F5* (Factor V Leiden), which likely contributes to the thrombotic signature of PAD and the benefit that patients receive by factor Xa inhibition.^21^ These genetic differences may play an important role in the predisposition of cancer in PAD.

In conclusion, the increased risk of major adverse cardiovascular events among patients with PAD relative to other forms of vascular disease is now well-accepted and has resulted in a significant effort to aggressively mitigate cardiovascular risk in this high-risk population. However, CVD only explains a portion of the increased mortality among patients with PAD and emerging evidence has implicated cancer as a key contributing factor. Our data suggest that patients who have been diagnosed with PAD may benefit from stringent adherence to cancer screening guidelines and further supports a direct link between the pathogenesis of atherosclerotic diseases and cancer.

### Study Limitations

The study design was formulated to rigorously test the central hypothesis, but the data and analyses have limitations. As a retrospective observational study, these data are unable to establish a causal link between PAD and cancer but may be clinically informative for the assessing the risk of cancer among patients with PAD. The analysis controlled for known sources of confounding including age, sex, diabetes, hypertension, hyperlipidemia, chronic kidney disease, smoking, insurance status, and geographic region; however, residual unknown confounders may remain. Biases may have been introduced by increased health care contact among the CVD cohorts and relies on the accuracy of incident cancer reporting, which can be subject to misclassification in claims data. Furthermore, ICD codes are only applied when disease is noted clinically and therefore inherently under-represents the true burden of disease, which would be expected in often subclinical diseases like PAD. Similarly, determination of tobacco use with ICD coding in claims data has known limitations, including underascertainment. Therefore, smoking may play a larger role than depicted in our analyses. We observed evidence of nonproportionality when assessing the subdistribution hazards model. However, the modeled cumulative incidence functions were ordered without overlap and PAD was consistently associated with the highest cumulative incidence of cancer throughout follow-up. Regardless, the reported subdistribution HR should be conservatively interpreted as an average association over follow-up rather than as a constant relative effect at every time point.

Perspectives **COMPETENCY IN MEDICAL KNOWLEDGE:** Patients with peripheral artery disease have higher rates of both cardiovascular and non-cardiovascular mortality. Our study identifies an increased incidence of several cancer subtypes among patients with PAD, underscoring the importance of comprehensive preventive care. These findings may support shared decision-making and reinforce adherence to existing guideline-recommended cancer screening.

**TRANSLATIONAL OUTLOOK:** Our study adds to a growing body of literature suggesting a bidirectional relationship between cancer and cardiovascular disease. Recent mechanistic studies have identified shared dysregulated pathways that contribute to both malignancy and cardiovascular pathology. Further investigation of these common biological processes may reveal novel therapeutic strategies with the potential to reduce the burden of both cancer and cardiovascular disease.

## Funding support and author disclosures

This research was supported, in part, by grant No. CCSG P30 CA016672 from the 10.13039/100000002National Institutes of Health. Dr Nead is supported by grant RP250062 from the Cancer Prevention and Research Institute of Texas (10.13039/100004917CPRIT) and grant K08CA263313 from the 10.13039/100000054National Cancer Institute. Dr Leeper is supported by grant numbers R35HL 144475 and 176060 from the 10.13039/100000050National Heart Lung and Blood Institute. Dr Giordano is supported by Komen SAC 150061 and CPRIT RP210140. The authors have reported that they have no relationships relevant to the contents of this paper to disclose.
