# Supplementary material for: Disproportionate Risk of Cancer Following Diagnosis of Peripheral Artery Disease
Source: JACC Adv. 2026 Jun 15;5(7):102889. doi: 10.1016/j.jacadv.2026.102889 (PMC13285827; doi:10.1016/j.jacadv.2026.102889)
Supplement: Supplemental Tables 1 to 6 [file mmc1.docx]

Supplemental Tables:

sTable 1: Demographics of patients with and without mortality data

| Characteristic | No Mortality | Mortality | ChiSq p-value |
| --- | --- | --- | --- |
| Overall | 2184777 | 5309173 |  |
| Age (Mean ± SD) | 54.9 ± 10.2 | 56.4 ± 10.8 |  |
| Sex |  |  | <0.0001 |
| Male | 956344 (43.8%) | 2549046 (48.0%) |  |
| Female | 1228433 (56.2%) | 2760127 (52.0%) |  |
| Diabetes |  |  | <0.0001 |
| No | 1829956 (83.8%) | 4385413 (82.6%) |  |
| Yes | 354821 (16.2%) | 923760 (17.4%) |  |
| Hypertension |  |  | <0.0001 |
| No | 1368665 (62.6%) | 3159255 (59.5%) |  |
| Yes | 816112 (37.4%) | 2149918 (40.5%) |  |
| Hyperlipidemia |  |  | <0.0001 |
| No | 1330001 (60.9%) | 3070339 (57.8%) |  |
| Yes | 854776 (39.1%) | 2238834 (42.2%) |  |
| CKD |  |  | <0.0001 |
| No | 2144720 (98.2%) | 5199564 (97.9%) |  |
| Yes | 40057 (1.8%) | 109609 (2.1%) |  |
| ICD Smoking |  |  | <0.0001 |
| No | 1996967 (91.4%) | 4884657 (92.0%) |  |
| Yes | 187810 (8.6%) | 424516 (8.0%) |  |
| Insurance |  |  | <0.0001 |
| HMO | 336618 (15.4%) | 462912 (8.7%) |  |
| PPO | 1108016 (50.7%) | 2964186 (55.8%) |  |
| Other | 708724 (32.4%) | 1818720 (34.3%) |  |
| Unknown | 31419 (1.4%) | 63355 (1.2%) |  |
| Region |  |  | <0.0001 |
| Northeast | 459634 (21.0%) | 1008383 (19.0%) |  |
| North Central | 366256 (16.8%) | 1268420 (23.9%) |  |
| South | 933737 (42.7%) | 2264166 (42.6%) |  |
| West | 414483 (19.0%) | 751216 (14.1%) |  |
| Unknown | 10667 (0.5%) | 16988 (0.3%) |  |
| CVD |  |  | <0.0001 |
| PAD | 26765 (1.2%) | 87594 (1.6%) |  |
| Other CVD | 343256 (15.7%) | 917680 (17.3%) |  |
| None | 1814756 (83.1%) | 4303899 (81.1%) |  |

sTable 2. Diagnosis and procedure codes to define cardiovascular diseases, other comorbidities and cancer.

| **Diagnosis / Procedure Codes** | |
| --- | --- |
| **Coronary artery disease** | *Diagnosis*  (ICD-9) 410.xx (acute myocardial infarction); 411.xx (other acute and subacute forms of ischemic heart disease); 412 (old myocardial infarction); 414.0x, 414.2 - 414.9 (coronary atherosclerosis); 429.7x (sequelae of myocardial infarction)  (ICD-10) I21.x (acute myocardial infarction; excluding I21.Ax); I22.x (STEMI/NSTEMI); I23.x (complications following STEMI/NSTEMI); I24.x (other acute ischemic heart disease); I25.x (chronic ischemic heart disease; excluding I25.3, I25.4x); Z95.5 (presence of coronary angioplasty implant and graft); Z98.61 (coronary angioplasty status).  T82.211, T82.211A, T82.211D, T82.211S, T82.212, T82.212A, T82.212D, T82.212S, T82.213, T82.213A, T82.213D, T82.213S, T82.218, T82.218A, T82.218D, T82.218S, Z951  *Procedure*  (ICD-9 CM) 0.66 (PCTA); 36.0x (removal of coronary artery obstruction);  36.1x (bypass anastomosis for heart revascularization); 36.2 (heart revascularization by arterial implants); 36.3x (other heart revascularization); 17.55 (transluminal coronary atherectomy).  (ICD-10 PCS Diagnosis) 0210xxx (coronary bypass, one artery); 0211xxx (coronary bypass, two arteries); 0212xxx (coronary bypass, three arteries); 0213xxx (coronary bypass, four arteries); 0270xxx (coronary dilation, one artery); 0271xxx (coronary dilation, two arteries); 0272xxx (coronary dilation, three arteries); 0273xxx (coronary dilation, four arteries) (CABG) B2020ZZ, B2021ZZ, B202YZZ, B2030ZZ, B2031ZZ, B203YZZ, B212010, B2120ZZ, B212110, B2121ZZ, B212Y10, B212YZZ, B213010, B2130ZZ, B213110, B2131ZZ, B213Y10, B213YZZ, B22300Z, B2230ZZ, B22310Z, B2231ZZ, B223Y0Z,  B223YZZ, B223Z2Z, B223ZZZ, B233Y0Z, B233YZZ, B233ZZZ (CPT) (PCI) 92920, 92921, 92924, 92925, 92928, 92929, 92933, 92934,  92937, 92938, 92941, 92943, 92944, C9600, C9601, C9602, C9603,  C9604, C9605, C9606, C9607, C9608  (CABG) 33510, 33511, 33512, 33513, 33514, 33516, 33517, 33518,  33519, 33520, 33521, 33522, 33523, 33525, 33528, 33530, 33533, 33534,  33535, 33536, 35600, 4110F, 75762, 75764, 75766, 75767, 93551, C9604,  C9605, G8158, G8159, G8160, G8161, G8162, G8163, G8164, G8165, G8166, G8167, G8170, G8171, G8172, G8497, G8544, G8573, G8574 |

| **Peripheral artery disease, aortic atherosclerosis** | *Diagnosis*  (ICD-9) 433.xx (occlusion and stenosis of precerebral arteries); 440.xx (atherosclerosis); 443.9 (peripheral vascular disease, unspecified) (ICD-10) I65.xx (occlusion and stenosis of precerebral arteries, not  resulting in cerebral infarction); I70.xxx (atherosclerosis); I73.9 (peripheral vascular disease, unspecified); I75.xxx (atheroembolism)  *Procedure*  Peripheral Revascularization (carotid and cerebral included):  (ICD-9 CM) 00.55 (insertion of drug eluting non-coronary stent); 00.63 (perc insertion of carotid artery stent); 00.64 (perc insertion of extracranial artery stent); 00.65 (perc insertion of intracranial artery stent); 17.53 (perc atherectomy extracranial vessel); 17.54 (perc atherectomy intracranial vessel); 17.56 (atherectomy of noncoronary vessel); 38.1x (endarterectomy); 39.25, 39.26, 39.29 (peripheral bypass grafting); 39.50 (Angioplasty of other non-coronary vessel(s)); 39.90 (Insertion of non- drug-eluting peripheral (non-coronary) vessel stent(s)).  (ICD-10 PCS) 047Cxxx; 047Dxxx; 047Exxx; 047Fxxx; 047Hxxx;  047Jxxx; 047Kxxx; 047Lxxx; 047Mxxx; 047Nxxx; 047Pxxx; 047Qxxx; 047Rxxx; 047Sxxx; 047Txxx; 047Uxxx; 047Vxxx; 047Wxxx; 047Yxxx (lower extremities, dilation). 04C0xxx; 04CCxxx; 04CDxxx; 04CExxx; 04CFxxx; 04CHxxx; 04CJxxx; 04CKxxx; 04CLxxx; 04CMxxx; 04CNxxx; 04CPxxx; 04CQxxx; 04CRxxx; 04CSxxx; 04CTxxx; 04CUxxx; 04CVxxx; 04CY (extirpation of lower extremity artery). 04100Jx; 041C0Jx; 041D0Jx; 041H0Jx; 041H0Jx; 041H0Kx; 041H4Jx; 041J0Jx; 041J4Jx;  041K0Jx; 041K0Zx; 041L09x; 041L0Kx; 041L0Zx; 041Mxxx; 041Nxxx;  041Sxxx; 041Txxx; 041Uxxx (bypass of lower extremity artery).  (CPT) Peripheral Revascularization (carotid and cerebral included) 37205, 37206, 37207, 37208, 37236, 37237, 37184, 37185, 37186, 35302, 35303,  35304, 35305, 35306, 35331, 35351, 35355, 35361, 35363, 35371, 35372,  35381, 35452, 35454, 35456, 35459, 35470, 35472, 35473, 35474, 35483,  35492, 35493, 35495, 35521, 35533, 35537, 35538, 35539, 35540, 35556,  35558, 35563, 35565, 35566, 35571, 35583, 35585, 35587, 35621, 35623,  35637, 35638, 35646, 35647, 35654, 35656, 35661, 35663, 35665, 35666,  35671, 35700, 35876, 35879, 35881, 35883, 35884, 37184, 37185, 37186,  37205, 37206, 37207, 37208, 0236T, 0237T, 0238T, 37225, 37224, 37227,  37226, 37222, 37223, 37220, 37221, 37229, 37228, 37231, 37230, 37233,  37232, 37235, 37234, 35548 |
| --- | --- |
| **Cerebral vascular disease** | (ICD-9) 433.xx (occlusion and stenosis of precerebral arteries); 434.x (occlusion and stenosis of cerebral arteries); 435.x (TIA); 437.x (cerebral atherosclerosis)  (ICD-10) G45.x (TIA); I63.xx (cerebral infarction); I65.xx (occlusion and stenosis of precerebral arteries, not resulting in cerebral infarction); I66.xx  (occlusion and stenosis of cerebral arteries, not resulting in cerebral infarction); I67.2 (cerebral atherosclerosis) |
| **Myocardial diseases** | (ICD-9) 398.xx (other rheumatic heart disease); 402.xx (hypertensive heart disease); 404.xx (hypertensive heart and chronic kidney disease); 415.0  (acute cor pulmonale); 416.8, 416.9 (chronic pulmonary heart disease);  422.xx (acute myocarditis); 425.xx (cardiomyopathy); 428.xx (heart failure); 429.0, 429.1, 429.3, 429.8x (other ill-defined heart diseases).  (ICD-10) I11.x (hypertensive heart disease); I13.x (hypertensive heart and chronic kidney disease); I25.3 (aneurysm of heart); I26.0x (pulmonary embolism with acute cor pulmonale); I27.xx (other pulmonary heart disease); I40.x (acute myocarditis); I42.x (cardiomyopathy); I43 (cardiomyopathy in diseases classified elsewhere); I50.xxx (heart failure); I51.xx (complications and ill-defined descriptions of heart disease); I52 (other heart disorders in diseases classified elsewhere). |

| **Pericardial diseases** | (ICD-9) 391 (chronic rheumatic pericarditis); 420.xx (acute pericarditis);  423.x (other diseases of the pericardium);  (ICD-10) I30.x (acute pericarditis); I31.x (other diseases of the pericardium); I32 (pericarditis in diseases classified elsewhere) |
| --- | --- |
| **Valvular heart diseases** | (ICD-9) 391.x (rheumatic fever with heart involvement); 394.x (diseases of the mitral valve); 395.x (diseases of the aortic valve); 396.x (diseases of the mitral and aortic valve); 397.x (diseases of other endocardial structures); 421.x (acute and subacute endocarditis); 424.xx (other diseases of the endocardium);  (ICD-10) I01.x (acute rheumatic fever w/ heart involvement); I05.x (rheumatic mitral valve disease); I06.x (rheumatic aortic valve disease); I07.x (rheumatic tricuspid valve disease); I08.x (multiple valve disease); I09.x (other rheumatic heart diseases); I33.x (acute and subacute endocarditis); I34.x (nonrheumatic mitral valve disorders); I35.x (nonrheumatic aortic valve disorders); I36.x (nonrheumatic tricuspid valve disorders); I37.x (nonrheumatic pulmonary valve disorders); I38 (endocarditis, valve unspecified); I39 (endocarditis and heart valve disorders in diseases classified elsewhere) |
| **Arrhythmias** | *Diagnosis*  (ICD-9) 426.xx (conduction disorders); 427.xx (cardiac dysrhythmias) (ICD-10) I44.xx (atrioventricular and left bundle branch block); I45.xx (other conduction disorders); I47.x (paroxysmal tachycardia); I48.xx (atrial fibrillation and flutter); I49.xx (other cardiac arrhythmias.  *Procedure*  (ICD-10) 02K8xxx (conduction mapping) |
| **Aortic Disease** | (ICD-9) 441.xx (aortic aneurysm and dissection), (ICD-10) I71.xx (aortic aneurysm and dissection) |
| **Congenital heart disease** | (ICD-9) 745.xx (bulbus cordis anomalies and anomalies of cardiac septal closure); 746.xx (other congenital anomalies of heart); 747.0x - 747.4x (other congenital anomalies of the circulatory system).  (ICD-10) Q20.x (congenital malformations of cardiac chambers and connections); Q21.x (congenital malformations of the cardiac septa); Q22.x (congenital malformations of the pulmonary and tricuspid valves); Q23.x (congenital malformations of the aortic and mitral valves); Q24.x (other congenital malformations of the heart); Q25.xx (congenital malformations of great arteries); Q26.x (congenital abnormalities of great veins) |
| **Other CAD** | (ICD-10) I25.4x (coronary artery aneurysm and dissection) |

| **Comorbidities** |  |
| --- | --- |
| Diabetes | (ICD-9) 249.xx (secondary diabetes); 250.xx (diabetes mellitus); 357.2 (diabetic polyneuropathy); 362.0x (diabetic retinopathy); 366.41 (diabetic cataract)  (ICD-10) E08.x (diabetes mellitus due to underlying condition), E09.x (drug or chemical-  induced diabetes mellitus), E10.x (type 1 diabetes mellitus), E11.x (type 2 diabetes mellitus), E13.x (other specified diabetes mellitus) E14.x (Unspecified diabetes mellitus) |
| Hypertension | (ICD-9) 401.x (essential hypertension); 403.xx (hypertensive chronic kidney disease); 405.xx (secondary hypertension)  (ICD-10) I10 (primary hypertension); I12.x (hypertensive chronic kidney disease); I15.x  (secondary hypertension) |
| Obesity | BMI as provided in MarketScan data (continuous variable) |
| Chronic Kidney Disease | (ICD-9) 585.3, 585.4  (ICD-10) N18.3x (CKD stage 3); N18.4 (CKD stage 4); N18.5 (CKD stage 5); N18.6 (ESRD) |
| Hyperlipidemia | (ICD-9) 272  (ICD-10) E78.xx (disorders of lipoprotein metabolism, excluding E78.7 and E78.8); E88.81  (metabolic syndrome) |
| Tobacco use | (ICD-9) 305.1, 649.0x, 989.84, V15.82 (ICD-10) F17.200, 099.33, T65.2, Z87.891 |
| **Cancer diagnosis** |  |
| Lip, oral cavity, pharynx (head and neck) | (ICD-9) 140.0, 140.1, 140.3, 140.4, 140.5, 140.6, 140.8, 140.9, 141.0, 141.1, 141.2, 141.3,  141.4, 141.5, 141.6, 141.8, 141.9, 142.0, 142.1, 142.2, 142.8, 142.9, 143.0, 143.1, 143.8,  143.9, 144.0, 144.1, 144.8, 144.9, 145.0, 145.1, 145.2, 145.3, 145.4, 145.5, 145.6, 145.8,  145.9, 146.0, 146.1, 146.2, 146.3, 146.4, 146.5, 146.6, 146.7, 146.8, 146.9, 147.0, 147.1,  147.2, 147.3, 147.8, 147.9, 148.0, 148.1, 148.2, 148.3, 148.8, 148.9, 149.0, 149.1, 149.8,  149.9  (ICD-10) C00.0, C00.1, C00.2, C00.3, C00.4, C00.5, C00.6, C00.7, C00.8, C00.9, C01,  C02.0, C02.1, C02.2, C02.3, C02.4, C02.8, C02.9, C03.0, C03.1, C03.9, C04.0, C04.1, C04.8, C04.9, C05.0, C05.1, C05.2, C05.8, C05.9, C06.0, C06.1, C06.2, C06.8, C06.9, C07, C08.0, C08.1, C08.9, C09.0, C09.1, C09.8, C09.9, C10.0, C10.1, C10.2, C10.3, C10.4, C10.8, C10.9, C11.0, C11.1, C11.2, C11.3, C11.8, C11.9, C12, C13.0, C13.1, C13.2, C13.8, C13.9, C14.0, C14.2, C14.8 |
| Esophagus | (ICD-9) 150.0, 150.1, 150.2, 150.3, 150.4, 150.5, 150.8, 150.9  (ICD-10) C15.3, C15.4, C15.5, C15.8, C15.9 |
| Stomach | (ICD-9) 151.0, 151.1, 151.2, 151.3, 151.4, 151.5, 151.6, 151.8, 151.9  (ICD-10) C16.0, C16.1, C16.2, C16.3, C16.4, C16.5, C16.6, C16.8, C16.9 |
| Small intestine | (ICD-9) 152.0, 152.1, 152.2, 152.3, 152.8, 152.9  (ICD-10) C17.0, C17.1, C17.2, C17.3, C17.8, C17.9, C26.0 |
| Colon | (ICD-9) 153.0, 153.1, 153.2, 153.3, 153.4, 153.5, 153.6, 153.7, 153.8, 153.9  (ICD-10) C18.0, C18.1, C18.2, C18.3, C18.4, C18.5, C18.6, C18.7, C18.8, C18.9 |
| Rectosigmoid, rectum, anus | (ICD-9) 154.0, 154.1, 154.2, 154.3, 154.8  (ICD-10) C19, C20, C21.0, C21.1, C21.2, C21.8 |
| Liver, gallbladder, spleen | (ICD-9) 155.0, 155.1, 155.2, 156.0, 156.1, 156.2, 156.8, 156.9, 159.0, 159.1 (ICD-10) C22.0, C22.1, C22.2, C22.3, C22.4, C22.7, C22.8, C22.9, C23, C24.0, C24.1,  C24.8, C24.9, C26.1, C26.9 |

| Pancreas | (ICD-9) 157.0, 157.1, 157.2, 157.3, 157.4, 157.8, 157.9  (ICD-10) C25.0, C25.1, C25.2, C25.3, C25.4, C25.7, C25.8, C25.9 |
| --- | --- |
| Nasal cavity, middle ear,  accessory sinuses | (ICD-9) 160.0, 160.1, 160.2, 160.3, 160.4, 160.5, 160.8, 160.9  (ICD-10) C30.0, C30.1, C31.0, C31.1, C31.2, C31.3, C31.8, C31.9 |
| Larynx, trachea | (ICD-9) 161.0, 161.1, 161.2, 161.3, 161.8, 161.9, 162.0  (ICD-10) C32.0, C32.1, C32.2, C32.3, C32.8, C32.9, C33 |
| Lung | (ICD-9) 162.2, 162.3, 162.4, 162.5, 162.8, 162.9, 165.0, 165.8, 165.9  (ICD-10) C34.0, C34.1, C34.2, C34.3, C34.8, C34.9, C39.0, C39.9 |
| Thymus, heart,  mediastinum, pleura | (ICD-9) 163.0, 163.1, 163.8, 163.9, 164.0, 164.1, 164.2, 164.3, 164.8, 164.9  (ICD-10) C37, C38.0, C38.1, C38.2, C38.3, C38.4, C38.8 |
| Bone and articular cartilage | (ICD-9) 170.0, 170.1, 170.2, 170.3, 170.4, 170.5, 170.6, 170.7, 170.8, 170.9  (ICD-10) C40.0, C40.1, C40.2, C40.3, C40.8, C40.9, C41.0, C41.1, C41.2, C41.3, C41.4, C41.9 |
| Melanoma | (ICD-9) 172.0, 172.1, 172.2, 172.3, 172.4, 172.5, 172.6, 172.7, 172.8, 172.9  (ICD-10) C43.0, C43.1, C43.2, C43.3, C43.4, C43.5, C43.6, C43.7, C43.8, C43.9 |
| Mesothelial and soft tissue | (ICD-9) 158.0, 158.8, 158.9, 171.0, 171.2, 171.3, 171.4, 171.5, 171.6, 171.7,  171.8, 171.9, 176.0, 176.1, 176.2, 176.3, 176.4, 176.5, 176.8, 176.9  (ICD-10) C45.0, C45.1, C45.2, C45.7, C45.9, C46.0, C46.1, C46.2, C46.3, C46.4, C46.5,  C46.7, C46.9, C47.0, C47.1, C47.2, C47.3, C47.4, C47.5, C47.6, C47.8, C47.9, C48.0, C48.1,  C48.2, C48.8, C49.0, C49.1, C49.2, C49.3, C49.4, C49.5, C49.6, C49.8, C49.9, C49.A |
| Breast | (ICD-9) 174.0, 174.2, 174.3, 174.4, 174.5, 174.6, 174.8, 174.9, 175.0, 175.9  (ICD-10) C50.0, C50.1, C50.2, C50.3, C50.4, C50.5, C50.6, C50.8, C50.9 |
| Uterine | (ICD-9) 179, 182.0, 182.1, 182.8  (ICD-10) C54.0, C54.1, C54.2, C54.3, C54.8, C54.9, C55 |
| Ovarian | (ICD-9) 183  (ICD-10) C56.1, C56.2, C56.9 |
| Female genital organs, other | (ICD-9) 180.0, 180.1, 180.8, 180.9, 181, 183.2, 183.3, 183.4, 183.5, 183.8, 183.9,  184.0, 184.1, 184.2, 184.3, 184.4, 184.8, 184.9  (ICD-10) C51.0, C51.1, C51.2, C51.8, C51.9, C52, C53.0, C53.1, C53.8, C53.9, C57.0, C57.1, C57.2, C57.3, C57.4, C57.7, C57.8, C57.9, C58 |
| Prostate | (ICD-9) 185  (ICD-10) C61, Z19.1, Z19.2 |
| Male genital organs, other | (ICD-9) 186.0, 186.9, 187.1, 187.2, 187.3, 187.4, 187.5, 187.6, 187.7, 187.8, 187.9 (ICD-10) C60.0, C60.1, C60.2, C60.8, C60.9, C62.0, C62.1, C62.9, C63.0, C63.1, C63.2,  C63.7, C63.8, C63.9 |
| Renal cell carcinoma | (ICD-9) 189.0, 189.1  (ICD-10) C64.1, C64.2, C64.9, C65.1, C65.2, C65.9, C66.1, C66.2, C66.9 |
| Bladder | (ICD-9) 188.0, 188.1, 188.2, 188.3, 188.4, 188.5, 188.6, 188.7, 188.8, 188.9  (ICD-10) C67.0 , C67.1, C67.2, C67.3, C67.4, C67.5, C67.6, C67.7, C67.8, C67.9 |
| Urinary organs, other | (ICD-9) 189.2, 189.3, 189.4, 189.8, 189.9  (ICD-10) C68.0, C68.1, C68.8, C68.9 |
| Eye and adnexa | (ICD-9) 190.0, 190.1, 190.2, 190.3, 190.4, 190.5, 190.6, 190.7, 190.8, 190.9  (ICD-10) C69.0, C69.1, C69.2, C69.3, C69.4, C69.5, C69.6, C69.8, C69.9 |
| Brain, meninges, nerves | (ICD-9) 191.0, 191.1, 191.2, 191.3, 191.4, 191.5, 191.6, 191.7, 191.8, 191.9,  192.0, 192.1, 192.2, 192.3, 192.8, 192.9  (ICD-10) C70.0, C70.1, C70.9, C71.0, C71.1, C71.2, C71.3, C71.4, C71.5, C71.6, C71.7, C71.8, C71.9, C72.0, C72.1, C72.2, C72.3, C72.4, C72.5, C72.9 |

| Thyroid, endocrine (other) | (ICD-9) 193, 194.0, 194.1, 194.3, 194.4, 194.5, 194.6, 194.8, 194.9  (ICD-10) C73, C74.0, C74.1, C74.9, C75.0, C75.1, C75.2, C75.3, C75.4, C75.5, C75.8, C75.9 |
| --- | --- |
| Neuroendocrine tumors | (ICD-9) 209.0, 209.1, 209.2, 209.3, 209.4, 209.5, 209.6, 209.7  (ICD-10) C7A.0, C7A.1, C7A.8, C7B.0, C7B.1, C7B.8 |
| Lymphoma (B and T), plasma cell dyscrasias | (ICD-9) 200.0, 200.1, 200.2, 200.3, 200.4, 200.5, 200.6, 200.7, 200.8, 201.0,  201.1, 201.2, 201.4, 201.5, 201.6, 201.7, 201.9, 202.0, 202.1, 202.2, 202.3, 202.4, 202.5,  202.7, 202.8, 202.9, 203.0, 203.1  (ICD-10) C81.0, C81.1, C81.2, C81.3, C81.4, C81.7, C81.9, C82.0, C82.1, C82.2, C82.3,  C82.4, C82.5, C82.6, C82.8, C82.9, C83.0, C83.1, C83.3, C83.5, C83.7, C83.8, C83.9, C84.0,  C84.1, C84.4, C84.6, C84.7, C84.A, C84.Z, C84.9, C85.1, C85.2, C85.8, C85.9, C86.0,  C86.1, C86.2, C86.3, C86.4, C86.5, C86.6, C88.0, C88.2, C88.3, C88.4, C88.8, C88.9, C90.0, C90.1, C90.2, C90.3 |
| Leukemia (lymphoid, myeloid, and other) | (ICD-9) 204.0, 204.1, 204.2, 204.8, 204.9, 205.0, 205.1, 205.2, 205.3, 205.8,  205.9, 206.0, 206.1, 206.2, 206.8, 206.9, 207.0, 207.1, 207.2, 207.8, 208.0, 208.1, 208.2,  208.8, 208.9  (ICD-10) C91.0, C91.1, C91.3, C91.4, C91.5, C91.6, C91.A, C91.Z, C91.9, C92.0, C92.1,  C92.2, C92.3, C92.4, C92.5, C92.6, C92.A, C92.Z, C92.9, C93.0, C93.1, C93.3, C93.Z,  C93.9, C94.0, C94.2, C94.3, C94.4, C94.6, C94.8, C95.0, C95.1, C95.9 |
| Hematologic malignancies,  other | (ICD-9) 202.6, 203.8, 238.4, 238.7  (ICD-10) C96.0, C96.2, C96.4, C96.5, C96.6, C96.A, C96.Z, C96.9, D45, D46 |
| Personal history of malignancy (only used for exclusion but not for incident  cancer) | (ICD-9) V10.x (excluding V10.83), V87.41, V87.43 (ICD-10) Z85.x (excluding Z85.82), Z86.00x, Z92.21, Z92.23 |

ICD, International Classification of Disease; CM, Clinical Modification; PCS, Procedure Coding System; CPT, Current Procedural Terminology; CVD, cardiovascular disease; STEMI, ST- elevation myocardial infarction; NSTEMI, non-ST-elevation myocardial infarction; PCI, percutaneous coronary intervention; PCTA, percutaneous transluminal coronary angioplasty; CABG, coronary artery bypass grafting; CKD, chronic kidney disease; ESRD, end-stage renal disease.

sTable 3: Standardized mean differences before and after weighting

| **Predictor** | **PAD vs. No PAD** | |
| --- | --- | --- |
|  | Unweighted | Weighted |
| **Continuous Age** | 1.564 | 0.178 |
| **Sex** | 0.038 | 0.158 |
| **Hypertension** | 1.173 | 0.036 |
| **Hyperlipidemia** | 0.862 | 0.032 |
| **Diabetes** | 0.900 | 0.009 |
| **CKD** | 0.531 | 0.020 |
| **Smoking** | 0.457 | 0.012 |

sTable 4: Presentation of covariates between the three clinical cohorts after weighting

| Characteristic | PAD Cases | CVD Controls | No CVD Controls |
| --- | --- | --- | --- |
| Age (Mean ± SD) | 54.4 ± 11.8 | 56.0 ± 11.1 | 56.3 ± 10.6 |
| Sex |  |  |  |
| Male | 37277 (39.4%) | 406318 (44.5%) | 2055192 (47.8%) |
| Female | 57380 (60.6%) | 507686 (55.5%) | 2246789 (52.2%) |
| Diabetes |  |  |  |
| No | 77622 (82.0%) | 744331 (81.4%) | 3550514 (82.5%) |
| Yes | 17035 (18.0%) | 169673 (18.6%) | 751467 (17.5%) |
| Hypertension |  |  |  |
| No | 57722 (61.0%) | 528768 (57.9%) | 2559046 (59.5%) |
| Yes | 36935 (39.0%) | 385236 (42.1%) | 1742935 (40.5%) |
| Hyperlipidemia |  |  |  |
| No | 55867 (59.0%) | 511433 (56.0%) | 2484476 (57.8%) |
| Yes | 38790 (41.0%) | 402571 (44.0%) | 1817505 (42.2%) |
| CKD |  |  |  |
| No | 92944 (98.2%) | 894666 (97.9%) | 4212780 (97.9%) |
| Yes | 1713 (1.8%) | 19338 (2.1%) | 89201 (2.1%) |
| ICD Smoking |  |  |  |
| No | 87328 (92.3%) | 838043 (91.7%) | 3957543 (92.0%) |
| Yes | 7329 (7.7%) | 75960 (8.3%) | 344438 (8.0%) |
| Insurance |  |  |  |
| HMO | 6419 (6.8%) | 75118 (8.2%) | 384609 (8.9%) |
| PPO | 54266 (57.3%) | 529635 (57.9%) | 2385883 (55.5%) |
| Other | 31766 (33.6%) | 296336 (32.4%) | 1481168 (34.4%) |
| Unknown | 2205 (2.3%) | 12914 (1.4%) | 50321 (1.2%) |
| Region |  |  |  |
| Northeast | 25847 (27.3%) | 217844 (23.8%) | 776784 (18.1%) |
| North Central | 21117 (22.3%) | 208435 (22.8%) | 1030090 (23.9%) |
| South | 40870 (43.2%) | 379619 (41.5%) | 1850860 (43.0%) |
| West | 6429 (6.8%) | 105185 (11.5%) | 630427 (14.7%) |
| Unknown | 394 (0.4%) | 2921 (0.3%) | 13820 (0.3%) |

sTable 5: Median follow up time and 5-year cumulative cancer incidence

| Group | Median Follow-Up Time [IQR] | 5 Year Cumulative Incidence of Cancer |
| --- | --- | --- |
| Overall | 31 [16 – 42] |  |
| No CVD | 31 [17 – 43] | 9.24% (9.19 – 9.28) |
| CVD | 32 [15 – 40] | 15.11% (15.00 – 15.23) |
| PAD | 31 [14 – 40] | 18.84% (18.47 – 19.20) |

STable 6: Quantitative bias assessment for tobacco use prevalence


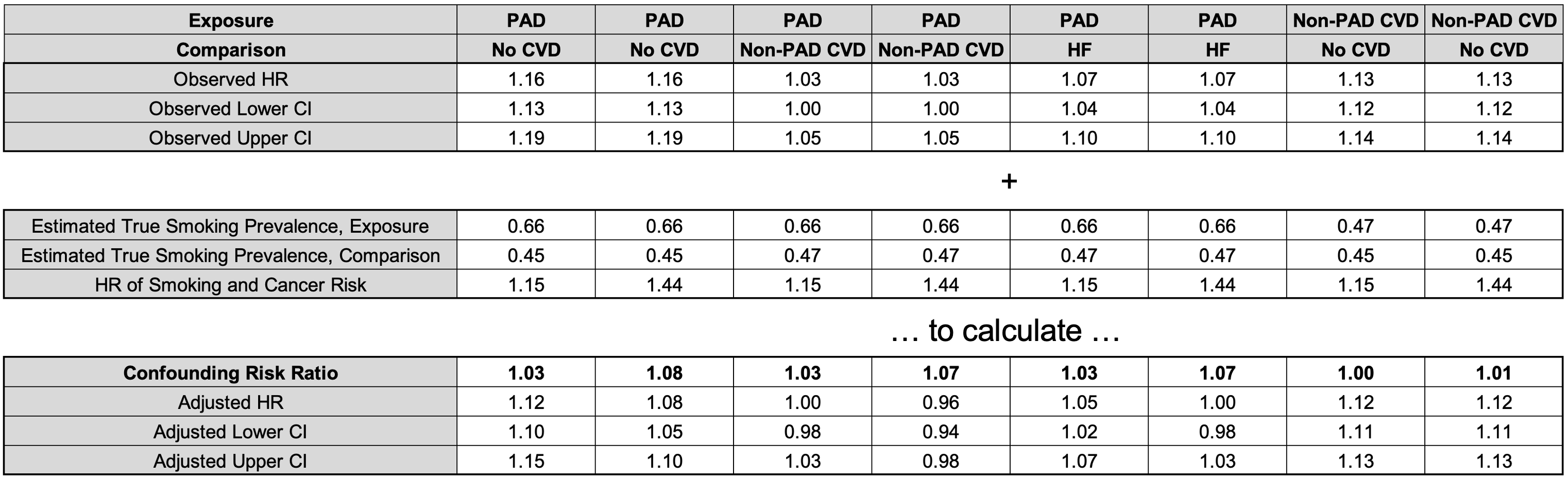


For each pair of exposure and comparator, the estimated prevalence of smoking for each group as well as the expected HR of smoking and cancer risk was identified from the literature (references 13-15). This information was used to calculate the confounding risk ratio and adjusted hazard ratios.
